# Platelet concentrate as an additive to bone allografts: a laboratory study using an uniaxial compression test

**DOI:** 10.1007/s10561-018-9704-3

**Published:** 2018-05-31

**Authors:** David Putzer, Markus Dobersberger, Alex Pizzini, Debora Coraça-Huber, Christoph Ammann, Michael Nogler

**Affiliations:** 1 Department of Orthopaedics – Experimental Orthopaedics, Medical University of Innsbruck, Innrain 36, 6020 Innsbruck, Austria; 2 Department of Internal Medicine, Medical University of Innsbruck, Anichstrasse 35, 6020 Innsbruck, Austria

**Keywords:** Processing allografts, Platelet concentrate, Additive to allografts, Chemical cleaning procedures, Platelet concentrate gel

## Abstract

Chemical cleaning procedures of allografts are destroying viable bone cells and denaturing osteoconductive and osteoinductive proteins present in the graft. The aim of the study was to investigate the mechanical differences of chemical cleaned allografts by adding blood, clotted blood; platelet concentrate and platelet gel using a uniaxial compression test. The allografts were chemically cleaned, dried and standardized according to their grain size distribution. Uniaxial compression test was carried out for the four groups before and after compacting the allografts. No statistically significant difference was found between native allografts, allografts mixed with blood, clotted blood, platelet concentrate and platelet concentrate gel regarding their yield limit after compaction. The authors recommend to chemical clean allografts for large defects, optimize their grain size distribution and add platelet concentrate or platelet rich plasma for enhancing as well primary stability as well bone ingrowth.

## Introduction

Bone grafts are used to fill bone defects in different applications of orthopaedic and trauma surgery with good long term results (Schreurs et al. 2009). Autografts are the gold standard in reconstructive surgery; however they are available only in limited quantity. They can be obtained from the femoral head during total hip arthroplasty or from the iliac crest (Khan et al. 2005; Myeroff and Archdeacon 2011; Nogler et al. 2012). Autografts have optimum osteoconductive, osteoinductive properties and allow osteogenesis as they contain surviving cells and osteoinductive proteins (BMPs) such as BMP-2 and BMP-7, fibroblast growth factor (FGF), insulin-like growth factor (IGF) and platelet-derived growth factor (PDGF) (Bauer and Muschler 2000; Dimitriou et al. 2011; Brydone et al. 2010; Parikh 2002).

To compensate for the reduced availability of autografts, allografts or synthetic materials are widely used. Allografts have variable osteoinductive and osteoconductive properties but are lacking viable cells which results in lower osteogenic potential than autografts (Zimmermann and Moghaddam 2011).

Sterilization processing of allografts includes the usage of hypotonic solutions, acetone, ethylene oxide, or gamma irradiation which can eliminate cellular and viral particles and therefore reduce the risk of infectious and transmissible diseases (Muller et al. 2013). Chemical cleaning processes are also used to remove the fat content of the allografts enhancing the mechanical properties of the allograft (Putzer et al. 2014a; van der Donk et al. 2003; Fosse et al. 2006; Voor et al. 2004). However, despite modern sterilization and storage methods, processing of allografts is not completely safe (Zimmermann and Moghaddam 2011; Malinin and Temple 2007).

The chemical cleaning as well as gamma irradiation of allografts may destroy the bone cells and denature proteins present in the graft and alter osteoconductive and osteoinductive characteristics, essentially eliminating the osteogenic properties and inhibiting the bone remodeling process (Keating and McQueen 2001).

During fracture healing and implant ingrowth the recruitment and migration of osteogenic cells are essential for bone regeneration (Kark et al. 2006). The migration of these cells is stimulated by growth factors as transforming growth factors (TFG), PDGF, IGF, vascular endothelial growth factors (VEGF), platelet derived angiogenic factor (PDAF) and FGF (Fiedler et al. 2002; Mayr-Wohlfart et al. 2002; Martineau et al. 2004; Wang and Avila 2007), all of which are released by platelets in response to injury (Martineau et al. 2004; Weibrich et al. 2002). In addition to growth factors (GFs), platelets release numerous other substances (e.g., fibronectin, vitronectin, sphingosine 1-phosphate, etc.…) that are important in wound healing (Wang and Avila 2007). Platelets can be applied as autologous product to sites of bone injury by either being concentrated in combination with blood plasma [platelet-rich plasma (PRP)] or as a platelet gel that is created by clotting the PRP (Kark et al. 2006).

PRP, plasma rich in growth factors (PRGF), and platelet concentrate (PLC) are essentially an increased concentration of autologous platelets suspended in a small amount of plasma after centrifugation (Wang and Avila 2007). By centrifugation donors blood is separated into platelet poor plasma (PPP), PRP and red blood cells (Marlovits et al. 2004). Prior to application, topical bovine thrombin and 10% calcium chloride is added to activate the clotting cascade, producing a 3–5 times higher platelet concentrated gel than native plasma contains (Petrungaro 2001). The release of PDGF, IGF, VEGF, PDAF and TGF-β present in the PRP is triggered by the activation of platelets by means of a variety of substances or stimuli such as thrombin, calcium chloride, or collagen (Wang and Avila 2007).

PRP indeed is widely used in plastic surgery and jaw reconstruction surgery for enhancing bone and connective tissue growth (Wang and Avila 2007; Marx et al. 1998; Board et al. 2008; Anitua et al. 2004; Thorwarth et al. 2006). Enriched platelet preparations have shown a rapid bone healing and regeneration when combined with autologous bone and bone substitute materials (Anitua et al. 2004; Kim et al. 2001). In PRP no cross reactivity, immune reaction or disease transmission has been observed (Weibrich et al. 2001). A reduced healing time (50%) has been shown in a study of Kassolis and Reynolds (2005). In animal studies no statistically significant difference was found between cancellous bone graft material with and without PRP (Butterfield et al. 2005; Jakse et al. 2003).

There is large evidence that by adding blood, autologous PRP or PLC the bone in-growth is enhanced, although antigenity is getting reduced in some cases (Khan et al. 2005; Anitua et al. 2004; Hannink et al. 2009; Baylink et al. 1993; Canalis et al. 1989; Canalis 1985; Lozada et al. 2001; Cenni et al. 2010; Blair and Flaumenhaft 2009). The aim of the study was to investigate the mechanical differences of chemical cleaned allografts with known grain size distribution mixed with blood (BL), clotted blood (CB), platelet concentrate (PC) and platelet concentrated gel (PG) using an uniaxial compression test.

## Methods

Bone tissue was donated by 5 patients to the local bone bank, from whom informed consent was obtained. In the preparation of allografts the local bone bank requirements for producing fresh-frozen allografts were followed. Cartilage and cortical tissue was removed and using a bone mill (Spierings Medische Techniek BV, Nijmegen, The Netherlands) allografts sizing 5–10 mm were produced (McNamara 2010). The allografts were frozen to − 80 °C, carefully mixed to reduce patient specific properties and stored at − 11 °C. A chemical cleaning procedure was used to remove fat content of the allografts and reduce the contamination risk (Coraca-Huber et al. 2013). For the cleaning procedure a sonicator (40 kHz, 200 W_eff_, BactoSonic, Bandelin eletronic GmbH & Co. KG, Berlin, Germany) was used. As washing solutions 700 ml of 1% Triton X-100 (Sigma-Aldrich, Schnelldorf, Germany), 500 ml 3% hydrogen peroxide solution (Sigma-Aldrich, Schnelldorf, Germany) and a 70% ethanol solution were used. The allografts were dried in an incubator (Memmert GmbH & Co. KG, Schwabach, Germany) at 37 °C.

Allograft samples were assembled according to their grain size in proportions specified in Table 1 after being separated using sieves ranging from 0.063 to 16 mm in correspondence to ASTM C 125 standard (Application time 1 h, Amplitude 10 mm, Haver und Böcker, Ölde, Germany) (Putzer et al. 2014b). Samples with a mean weight of 8 ± 0.01 g were obtained and divided into four groups each containing 20 samples.

**Table 1:** All samples were reassembled after sieving with specific grain size proportions achieving a total weight of 8 g

Grain size [mm]	Weight [g]
> 4	5.08
4–2	0.86
2–1	0.60
< 1	1.46
Total	8.00

In one group (BL) 4 ml blood from the same donor, who gave his informed consent prior donation, were added. In different studies native allografts showed a liquid component of 50% in weight (Putzer et al. 2014a). Adding 4 ml of blood approximately compensates the liquid component, which was previously removed. The blood was stored at − 4 °C and was obtained from the local tissue bank. In the second group (CB) clotting was induced by adding 480 μl of 1 mol calcium chloride (CaCl_2_) in addition to the 4 ml blood and mixed thoroughly for 1 min as described by Oakley and Kuiper (2006) and Camenzind et al. (2000). The cleaned allografts were mixed with the clotted blood and after 5 min (6 min activation time) the samples were used for mechanical testing.

In group PC 4 ml of concentrated platelets from one donor, who gave his informed consent prior donation, were added. The PC was stored at − 4 °C. In platelet concentrated gel (PG) in addition to the 4 ml of concentrated platelets 666 μl of 1 mol calcium chloride (CaCl_2_) were added (Marx et al. 1998; Oakley and Kuiper 2006; Camenzind et al. 2000). After 6 min activation time the samples were used for mechanical testing.

All samples were filled into a compaction chamber with an internal diameter of 40 mm. A uniaxial compression test was carried out before and after a standardized compaction procedure resulting in 20 measurements before and 20 measurements after compaction for each of the four groups. In the standardized compaction procedure a fall hammer (1.45 kg) was dropped 10 times from a height of 18 cm.

The uniaxial compression test was carried out with a 15 mm punch which was lowered with a speed of 1 mm/min into the allografts. Force and displacement were measured by the testing machine after reaching a preload of 5 N (Zwicki—Line Z 2.5, maximal load 2.5 kN, 320 kHz sample rate, Zwick GmbH & Co. KG, Ulm, Deutschland) with a preciseness of ± 0.04 N and ± 2 μm.

**Fig. 1:**
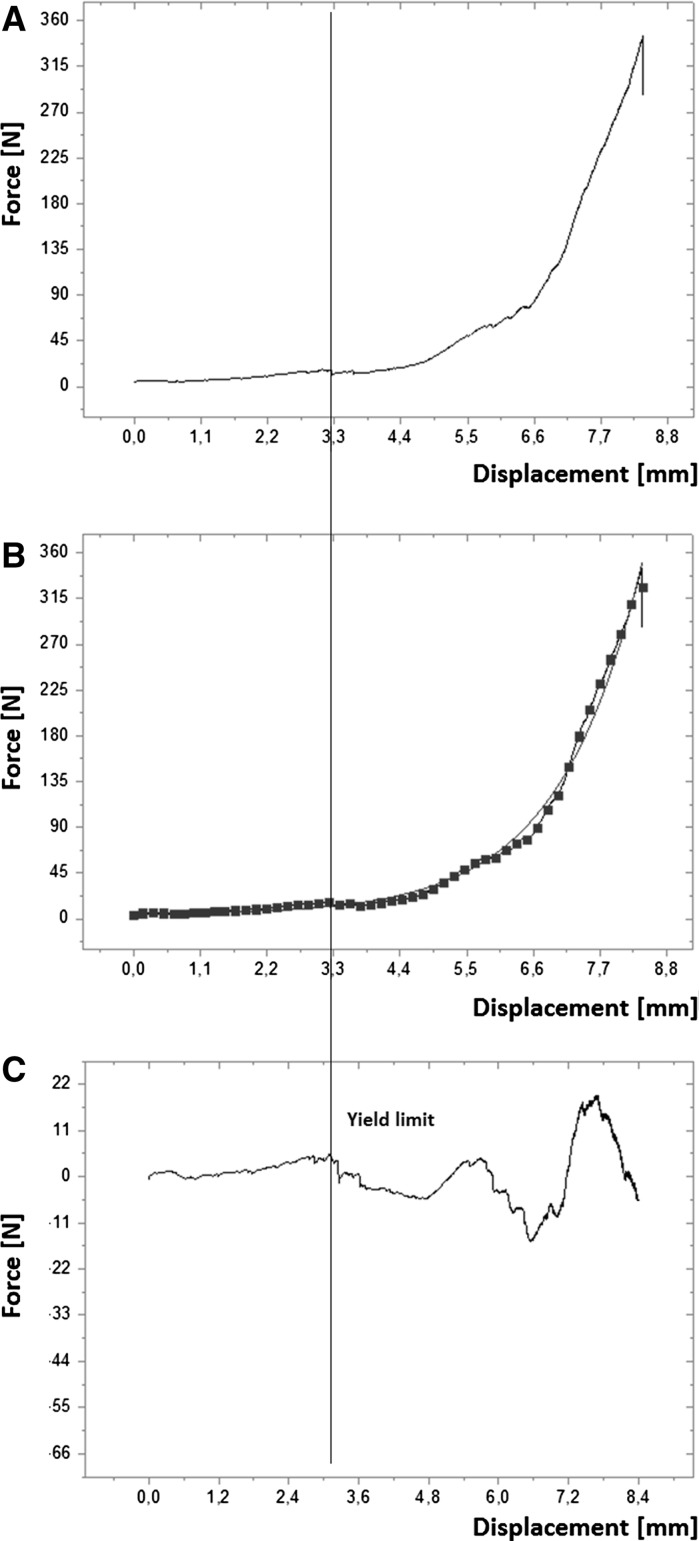
**a** Force displacement curves were from uniaxial compression test using a testing machine. **b** In all curves a fitted baseline with 50 anchor points (squares) was subtracted to remove the logarithmic trend. **c** The signal was analyzed for positive local maxima. The yield limit (YL) was determined as the first local maxima on the force curve and is indicated by a line in all three graphs

A peak analysis was performed on the resulting force displacement curves using OriginPro8.5 (Origin Lab Corporation, Northampton, Massachusetts, USA) (Fig. 1a). In all curves a fitted baseline (50 anchor points, 1 and 2 derivation method, polynomial smoothing of order 2) was subtracted to remove the logarithmic trend (Fig. 1b). The signal was analyzed for positive local maxima over 100 data points with a smoothing window size of 10 data points. The yield limit (YL) was determined as the first local maxima on the force curve (Fig. 1c). The corresponding displacement value was used to obtain the density at the yield limit δ_YL_. The punch displacement value at 5 N was used to calculate the initial density of the samples δ_i_.

Previously published data by the authors were used as control groups were native allografts (NA) (Putzer et al. 2014a) and allografts with optimized grain size distribution (OG) were evaluated (Putzer et al. 2014b).

Measurements of each variable and group were tested for normal distribution using the Kolmogorov–Smirnov Test. Comparison before and after compaction were analyzed using the two-tailed *T* Test for dependent samples. All groups were tested for variance homogeneity. In case of variance homogeneity ANOVA was used for group comparisons and Games Howel Post-Hoc analysis for pairwise comparisons. If variance homogeneity was not fulfilled the Brown Forsythe test was used for group comparisons and Tukey Post-Hoc analysis was used for pairwise comparison. Outliers were defined as values that are greater than 100% and smaller than 10% of the median. They were removed from the data set. In all analysis (SPSS software v.20, IBM, Chicago, IL) a *p* value of 0.05 was considered statistically significant.

## Results

NA, OG, BL, PC and PG had a normal distribution for all investigated measurement parameters. CB showed a normal distribution except for the yield limit before compaction. IN BL 1 outlier was eliminated for the yield limit before and 1 outlier after compaction. In CB one outlier was eliminated for the initial density, two outliers were eliminated for the density at the yield limit and two for the yield limit when uncompacted. After compaction in CB one outlier was eliminated for the yield limit. PC showed two outliers for the initial density when uncompacted. PG showed two outliers for the yield limit before compaction.

No statistically significant change of the initial density was observed after compaction for BL and PC (Table 2). In NA a statistically significant increase of 22% and in OG a of 34% was observed, while CB showed a statistical significant increase of the initial density by 10% and PG increased its initial density after compaction by 13%.

**Table 2:** Mean and standard deviation of the initial density before and after compaction are reported for the four groups under investigation

δ_i_ [g/cm^3^]	Uncompacted	Compacted	Difference	*p* value
NA	0.91 (SD 0.14)	1.17 (SD 0.09)	22.3	< 0.001
OG^a^	0.39 (SD 0.04)	0.59 (SD 0.07)	33.9	< 0.001
BL	1.12 (SD 0.08)	1.14 (SD 0.22)		0.695
CB	1.13 (SD 0.09)	1.26 (SD 0.30)	10.3	0.044
PC	1.13 (SD 0.09)	1.14 (SD 0.14)		0.621
PG	1.16 (SD 0.08)	1.34 (SD 0.26)	13.4	0.004

The difference was calculated as a percentage and *p* value of the *T* test (comparison before and after compaction) is reported

^a^The sample weight was half of the other groups

Considering the density at the yield limit before and after compaction BL showed a statistically significant increase of 13% and PG of 14%, while NA showed an increase of 10% and OG of 22% (Table 3). In CB and PC no statistically significant increase of the density at the yield limit could be observed.

**Table 3:** Mean and standard deviation of the density at the yield limit before and after compaction are reported for the four groups under investigation

δ_YL_ [g/cm^3^]	Uncompacted	Compacted	Difference	*p* value
NA	1.45 (SD 0.24)	1.60 (SD 0.21)	10.1	0.008
OG^a^	0.58 (SD 0.19)	0.74 (SD 0.08)	21.7	< 0.001
BL	1.48 (SD 0.13)	1.71 (SD 0.46)	13.4	0.049
CB	1.48 (SD 0.13)	1.48 (SD 0.30)		0.969
PC	1.53 (SD 0.19)	1.70 (SD 0.35)		0.178
PG	1.53 (SD 0.13)	1.78 (SD 0.45)	14.0	0.031

The difference was calculated as a percentage and *p* value of the *T* test (comparison before and after compaction) is reported

^a^The sample weight was half of the other groups

All groups showed a statistical significant difference when comparing the yield limit before and after compaction (Table 4). BL and PC showed a ~ 35% higher yield limit after compaction, while in the groups with the activation liquid CB and PG the yield limit increased by 15% for CB and 20% for PG. NA showed an increase of the yield limit by 80% and OG of 90%.

**Table 4:** Mean and standard deviation of the yield limit before and after compaction are reported for the four groups under investigation

YL [kPa]	Uncompacted	Compacted	Difference	*p* value
NA	24 (SD 19)	117 (SD 62)	79.1	< 0.001
OG	35 (SD 37)	353 (SD 187)	90.1	< 0.001
BL	33 (SD 30)	90 (SD 28)	36.7	< 0.001
CB	12 (SD 14)	83 (SD 32)	14.4	< 0.001
PC	32 (SD 23)	91 (SD 32)	35.2	< 0.001
PG	16 (SD 11)	78 (SD 34)	20.5	< 0.001

The difference was calculated as a percentage and *p* value of the *T* test (comparison before and after compaction) is reported

The uncompacted initial density and uncompacted yield limit showed a homogeneity distribution of variances. All other variables did not show homogeneity of variances.

The OG group showed a statistically significant lower initial density and a lower density at the yield limit to all other groups before and after compaction (NA, BL, PG, CB and PC) (*p* ≤ 0.006). NA showed a statistically lower initial density to BL, PG, CB and PC (*p* ≤ 0.006) before compaction. All other groups (BL, PG, CB and PC) showed no statistically significant difference between each other (*p* > 0.376). After compaction no statistically significance was found for all pairwise comparison between NA, BL, CB, PC and PG (*p* > 0.105) except for a higher initial density which was found for PG in comparison to BL (*p* = 0.030).

All pairwise comparison between NA, BL, CB, PC and PG of the density at the yield limit before and after compaction did not reach statistical significance level (*p* > 0.080).

When considering the yield limit a statistically significant lower value could be found for CB in comparison to PC (*p* = 0.027), to NA (*p* = 0.008), to BL (*p* = 0.016) and to OG (*p* = 0.003) before compaction. After compaction OG showed a statistical significant higher yield limit in comparison to other groups (*p* ≤ 0.001). NA showed a statistically higher yield limit in comparison to PG (*p* = 0.038) after compaction. All other pairwise comparisons between groups did not reach statistically significance level (*p* > 0.077) as well before as after compaction.

## Discussion

Adding blood, PRP or PLC in allografts has shown in different studies to enhance bone ingrowth (Khan et al. 2005; Anitua et al. 2004; Hannink et al. 2009; Baylink et al. 1993; Canalis et al. 1989; Canalis 1985; Lozada et al. 2001; Cenni et al. 2010; Blair and Flaumenhaft 2009). It is therefore a promising additive to chemical cleaned allografts, were growth factors may be washed out by the cleaning procedure itself. Our measurements showed that the yield limit of four different prepared allografts did not significantly differ from each other after compaction and did not differ in comparison to native allografts. However a statistically significant difference was found in comparison to dried allografts with optimized grain size distribution. This findings are in accordance with several other studies (Putzer et al. 2014a, b; Fosse et al. 2006; Voor et al. 2004).

OG showed a statistically significant lower initial density and a lower density at the yield limit before and after compaction in all cases, as in the preparation process no liquids were added resulting in a sample weight of 8 g. All samples from the other groups had a weight of 16 g. In CB and PG the initial density was increased by more than 10%, while the other two groups, where clotting was not activated, did not show a statistically significant difference. The activation of the clotting may have induced a better interlocking between particles for the uncompacted allografts. After compaction initial density was similar between all groups PG had the highest initial density after compaction, which was statistically different from BL. As PG can be considered as a gel it can be deduced that the gel may absorb better kinetic energy during the standardized compaction procedure and therefore reduce its volume not as much as BL.

When considering the density at the yield limit a statistically significant reduction before and after compaction was found for BL and PG, which was again higher than 10%. Between the five groups under investigation no statistically significant difference was found. When considering the mean of each group after compaction, they are surprisingly similar for BL,CB, PC,PG and NA, which could be an indication for the mixage with blood for BL, CB and NA and mixage with platelets in PC and PG. However no statistically evidence was found for this observation and the samples did not differ from native allografts.

The yield limit was improved in all cases significantly after compaction. In case of the clotted groups CB and PG the difference before and after compaction was less prominent < 21% than for the not activated groups BL and PC, where an increase of > 35% was observed. In the uncompacted group the Yield limit seemed to be higher for BL and PC than for CB and PG although no statistically significant difference between groups could be found. After compaction the yield limit of al four groups (BL, PC, CB and PG) reached similar values and no difference could be found. It can be deduced that the liquid phase is absorbed in the spongious allograft material and plays an inferior role on the mechanical properties. OG showed the highest yield limit after compaction, which shows the benefit in reducing liquid and fatty content for the improving mechanical interlocking of the particles. NA showed a statistically higher yield limit in comparison to PG (*p* = 0.038) after compaction, however all other pairwise comparisons between groups did not reach statistically significance level (*p* > 0.077) as well before as after compaction. This indicates that all four mixtures are similar to native bone. Their usage can be recommended, especially the platelet concentrate gel as it should contain the highest amount of GF, while having similar mechanical properties to native allografts.

Several studies show, that the fat and liquid content of allografts reduce the primary stability of allografts (Putzer et al. 2014a; Fosse et al. 2006; Voor et al. 2004). However the authors believe that by optimizing the grain size distribution (Putzer et al. 2014b), defatting the graft material by an appropriate cleaning procedure (Coraca-Huber et al. 2013; Wurm et al. 2016) and adding GF using platelet concentrate gel or PRP will enhance primary stability and speed up bone growth in.

To reduce patient specific properties all samples were carefully remixed before usage to reduce any biasing effect. A part of liquids may be lost during the compaction process, altering the sample composition during the measurements. In our experiments a large quantity of liquids (50% in weight) were added to compensate for any liquid loss during the measurements. Bone quality was not assessed radiological by the authors, however all sample where previously screened for osteoporosis according to the quality guidelines of the local bone bank.

## Conclusion

In conclusion the study shows that there was no statistically significant difference in the yield limit between allografts mixed with blood, clotted blood, platelet concentrate and platelet concentrate gel in comparison to native allografts. All of them are therefore suitable from a mechanical point of view to be used in bone impaction grafting to enhance bone remodeling by adding growth factors. From literature it seems that platelet concentrate gel or PRP has the highest change to speed up bone ingrowth. Adding liquids could decrease primary stability in comparison to dry allografts an optimum level of liquid content still needs to be defined. The authors recommend to chemical clean allografts for large defects, optimize their grain size distribution and add GF for enhancing bone ingrowth. All of the findings have to be evaluated and tested in an in vivo study for further applicability.
